# Generating segmentation masks of herbarium specimens and a data set for training segmentation models using deep learning

**DOI:** 10.1002/aps3.11352

**Published:** 2020-07-01

**Authors:** Alexander E. White, Rebecca B. Dikow, Makinnon Baugh, Abigail Jenkins, Paul B. Frandsen

**Affiliations:** ^1^ Data Science Lab Office of the Chief Information Officer Smithsonian Institution Washington D.C. USA; ^2^ Department of Botany National Museum of Natural History Smithsonian Institution Washington D.C. USA; ^3^ Department of Plant and Wildlife Sciences Brigham Young University Provo Utah USA

**Keywords:** deep learning, digitized herbarium specimens, ferns, machine learning, semantic segmentation, U‐Net

## Abstract

**Premise:**

Digitized images of herbarium specimens are highly diverse with many potential sources of visual noise and bias. The systematic removal of noise and minimization of bias must be achieved in order to generate biological insights based on the plants rather than the digitization and mounting practices involved. Here, we develop a workflow and data set of high‐resolution image masks to segment plant tissues in herbarium specimen images and remove background pixels using deep learning.

**Methods and Results:**

We generated 400 curated, high‐resolution masks of ferns using a combination of automatic and manual tools for image manipulation. We used those images to train a U‐Net‐style deep learning model for image segmentation, achieving a final Sørensen–Dice coefficient of 0.96. The resulting model can automatically, efficiently, and accurately segment massive data sets of digitized herbarium specimens, particularly for ferns.

**Conclusions:**

The application of deep learning in herbarium sciences requires transparent and systematic protocols for generating training data so that these labor‐intensive resources can be generalized to other deep learning applications. Segmentation ground‐truth masks are hard‐won data, and we share these data and the model openly in the hopes of furthering model training and transfer learning opportunities for broader herbarium applications.

Efforts to digitize natural history collections have resulted in large, open data sets that, when linked with collection data, offer an unprecedented opportunity to evaluate transformative research questions that link objects through space and time. The tens of millions of openly available digitized herbarium specimens allow botanists to evaluate ecological and evolutionary questions on a global scale, integrating across centuries of botanical collecting and piecing together collections with spatially biased holdings to generate biome‐level comparisons of biological patterns (Davis et al., 2015; Pearse et al., 2017; Park and Mazer, 2018). Collating millions of herbarium specimens into a single analysis, however, necessarily involves aggregating noise, error, and bias from thousands of different sampling, mounting, and digitization protocols, given that herbarium sheets are digitized in independent efforts in the approximately ~3000 herbaria worldwide (Thiers, 2018). Such bias and noise may influence not only the analysis of digitized collection records (e.g., Daru et al., 2018), but also analyses of specimen images, a growing source of data for botanical questions ranging from phenology (e.g., Willis et al., 2017; Lorieul et al., 2019) to morphology (e.g., Kavanagh et al., 2011; Burns et al., 2012; Easlon and Bloom, 2014) to global change biology (e.g., Meineke et al., 2018; Heberling et al., 2019). To move beyond the important efforts of quantifying sources of bias in the specimen records, we must therefore begin to use statistical methods that allow for the identification and elimination of sources of bias in image data to leverage images of herbarium specimens to their full potential.

Machine learning and its subfield of deep learning are particularly useful for the analysis of specimen images (Unger et al., 2016), as these types of models can be trained to identify and ignore sources of image variation and noise. For example, deep learning models in the form of convolutional neural networks (CNNs; LeCun et al., 2015) are well suited to classifying objects in images irrespective of the position or orientation of those objects. Although a variety of object orientations or contexts might pose a challenge to more traditional quantitative methods of classification, these sources of noise do not prevent deep learning models from achieving high accuracy for object identification. The ability of deep learning models to maintain both high classification accuracy and wide generality does not, however, preclude them from identifying a given image class (e.g., taxonomic identity) based on information that is otherwise non‐biological. Indeed, there are a number of visual cues that may allow a specimen to be identified based on the herbarium from which it originated rather than the features of the plant itself. Other biologically uninformative visual information may include rulers, a color bar or palette, collection‐specific barcodes, stamps, collection identifiers, text that might be included as standard in a given collection, or even accumulated sources of debris (e.g., mercuric chloride staining; see Schuettpelz et al., 2017). Although most of these visual cues may be easily interpreted as noise, which neural networks are well suited to ignore, the critical concern is unknown cases where these cues are unique, rare, and singularly associated with a specific class that the model is built to identify. These cues may undermine the performance of a deep learning model on a novel data set that lacks such cues. Systematic and scalable methods are needed to remove such cues from analyses that rely on digitized herbarium specimens as the primary source of data, particularly for deep learning data sets where the scale of the sample group often prohibits the manual inspection of each image.

One promising solution is to identify biologically relevant image pixels (i.e., plant tissues) a priori, eliminating noisy and biased visual information from all other parts of the herbarium specimen image ahead of subsequent analyses. Deep learning models can be trained to label image pixels based on their content (in this case, RGB values). Labeling image pixels in this way is referred to as *semantic segmentation*, and has been applied in other botanical applications, for example in automated agriculture (Milioto et al., 2018). Novel deep learning model architectures are also being developed to improve focal object attention during classification (e.g., attention‐based CNNs, Ba et al., 2014; Simonyan and Zisserman, 2014; Ren et al., 2015) and applied in the medical field (e.g., Li et al., 2019); however, there are also a number of potential neural network applications for these models in herbarium science beyond classification. In such applications (e.g., automated identification and measurement of specific plant tissues; Lorieul et al., 2019), the semantic segmentation of herbarium specimens may be critical for generating meaningful biological insights.

The primary and perhaps most critical step in developing an accurate deep learning model for semantic segmentation is to generate a high‐resolution data set of images with their associated *masks*, images of identical resolution that define the identity of each pixel in the original image as belonging to an a priori designated set of pixel identities or classes (e.g., plant tissue, label, color bar). These data (original images paired with their masks) are used to train the deep learning model to generate accurate pixel classifications by comparing the model predictions for each pixel against the pixel class defined in the mask (i.e., the *ground truth*). A more detailed description of the learning process is beyond the scope of this work, but see Garcia‐Garcia et al. (2017) for a general review of semantic segmentation and its applications in deep learning. Although image masks for herbarium specimens can be as simple as a binary image identifying two classes, one for all pixels containing plant tissues and another for all other visual information (background), herbarium specimens contain such a wide diversity of plant sizes and shapes that drawing boundaries along the edges of the specimens, particularly around complex leaf structures, is extremely labor intensive and nearly impossible on a large scale. Generating masks of complex plant structures at high resolution is not only labor intensive but also particularly difficult using polygon‐based annotation approaches such as those offered in ImageJ (Schneider et al., 2012; Rueden et al., 2017). In this paper, we present a systematic workflow for generating high‐quality image masks of digitized herbarium specimens for use with deep learning segmentation models. We describe our efforts to train and validate such a model using a Python‐based deep learning framework and share an open repository where the original images, ground‐truth masks, and the trained deep learning model can be accessed for future use outside the application we detail below.

Potential uses for the image segmentation of digitized herbarium images include the detection of flowers, the quantification of fruits, and the estimation of the intensity of disease and the extent of herbivory (e.g., Meineke and Davies, 2019); the data and model we present could be leveraged for those applications as well. Similar uses exist across different fields where machine learning has become an essential tool; for example, these types of analyses are vital in the medical field, where precision in the determination of tissue types and pathologies in medical images is essential for accurate diagnosis and successful treatment decisions made by medical professionals (e.g., Ciresan et al., 2012; Roth et al., 2015; Moeskops et al., 2016; Zhuang et al., 2019). There are other industrial applications of this technology beyond medicine, one of which, agriculture, is more closely related to the work we present here. For example, whereas high‐throughput genotyping has been used in plant breeding applications for decades, the nascent field of high‐throughput phenotyping is focused on generating reliable genotype–phenotype associations (Choudhury et al., 2019). The accurate evaluation of changes in phenotype requires models that result in well‐resolved measurements of phenotypic change (Singh et al., 2016).

Our workflow draws upon best practices and recent advances in computer vision and deep learning, making use of openly available Python libraries for image manipulation (OpenCV, Bradski and Kaehler, 2008; PlantCV, Gehan et al., 2017) and machine learning (PyTorch, Paszke et al., 2017; fastai, Howard et al., 2018). The deep learning model architecture we employ (U‐Net; Ronneberger et al., 2015) was originally developed in a medical context, but is now well known for its efficient and accurate performance in general image segmentation tasks. We combine these practices in one protocol to allow more systematic improvement and transparency regarding training data inputs in machine learning applications in herbarium science. Critically, to motivate model sharing and facilitate transfer learning applications within the herbarium science community, we share the trained model and these data, including both high‐resolution images and image masks, on Figshare (see Data Availability).

## MATERIALS AND RESULTS

### Data

**FIGURE 1:**
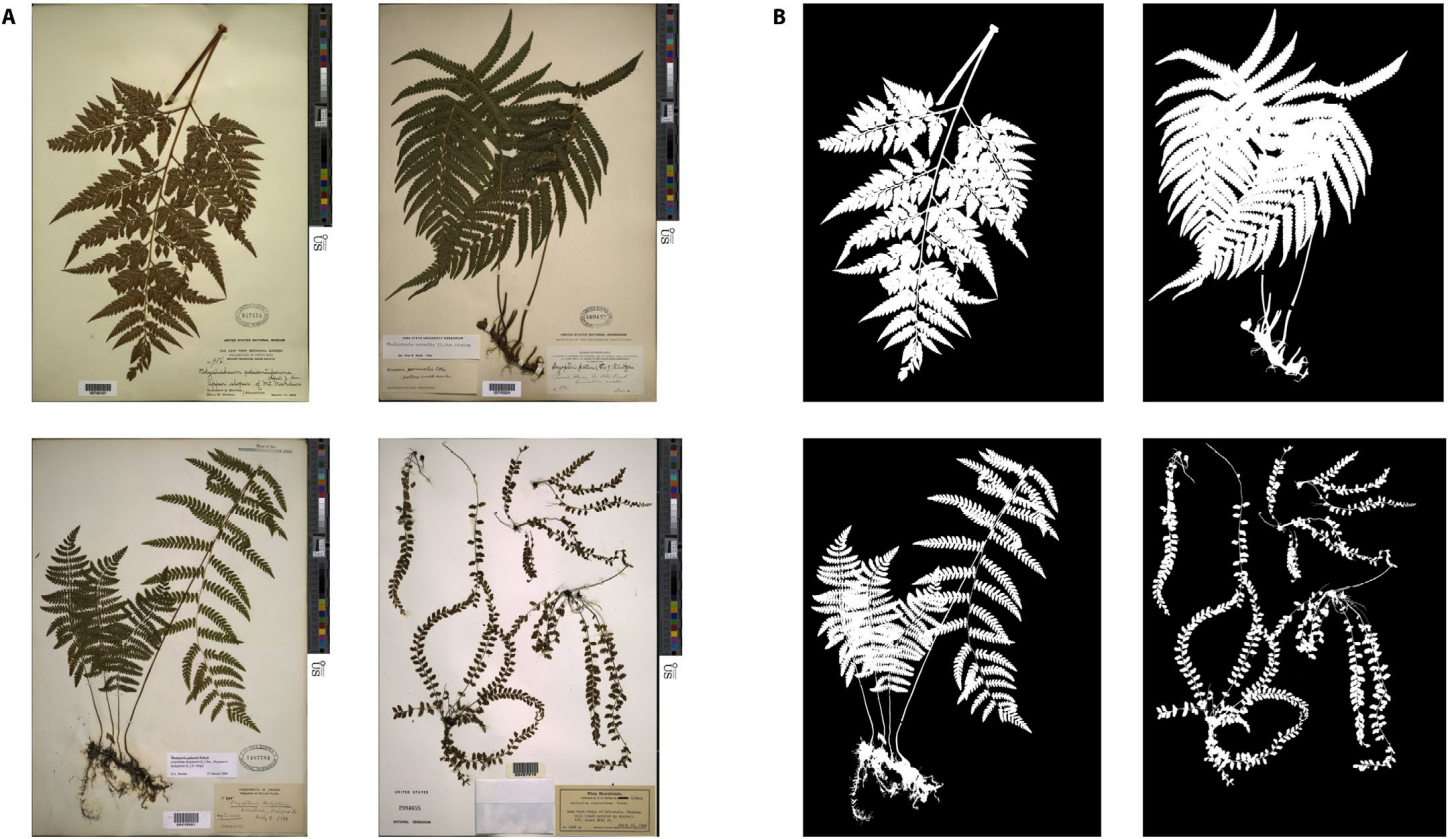
Herbarium sheets and associated masks made available in this study. (A) Four example digitized herbarium sheets from the U.S. National Herbarium at the National Museum of Natural History (Washington, D.C., USA). (B) The same four sheets shown as high‐resolution masks. A total of 400 masks were generated using the methods described in the text and were used to train a deep neural net to automatically segment plant tissues from herbarium specimens. Species names for each image clockwise from top left: *Rumohra adiantiformis* (G. Forst.) Ching, *Thelypteris kunthii* (Desv.) C. V. Morton (synonym *Christella kunthii*), *Asplenium peruvianum* var. *insulare* (C. V. Morton) D. D. Palmer, *Thelypteris palustris* Schott.

Our goal was to generate a segmentation model for plant tissues with complex margins, yet we felt that some measure of taxonomic specificity would increase the model’s accuracy and utility. Ferns are known to exhibit an incredible diversity of leaf forms (see Vasco et al. [2013] for a comprehensive review of fern leaf morphology) and, in the absence of fruits, flowers, and woody material, fern specimens in herbaria largely reflect leaf material mounted on paper. Recognizing both the broad diversity and complexity of fern leaf margins as well as their relatively simple representation across specimens within herbaria, we limited our data set to only include images of fern specimens. We obtained digital images (high‐resolution JPEG [.jpg] files, ca. 9100 × 6800 pixels) of all such specimens housed in the U.S. National Herbarium at the National Museum of Natural History (Washington, D.C., USA). The images were acquired from the Smithsonian Institution Digital Asset Management System and transferred to the Smithsonian High‐Performance Computing Cluster for analysis. To curate these images into a manageable data set for mask generation, we chose 400 random images and subjectively hand‐curated our image set to reflect the broad diversity of leaf shapes present in ferns as much as possible (Fig. 1A); replacement images were chosen at random until the set of images subjectively appeared to reflect the wide morphological diversity of ferns. Images without plant tissue (e.g., specimens housed in envelopes and affixed to sheets) were removed and replaced with another random specimen containing visible tissues. In total, the 400 images we curated include specimens from 11 orders, 30 families, 99 genera, and 308 species (specimen metadata is included, see Data Availability).

### Protocol for generating image masks

**FIGURE 2:**
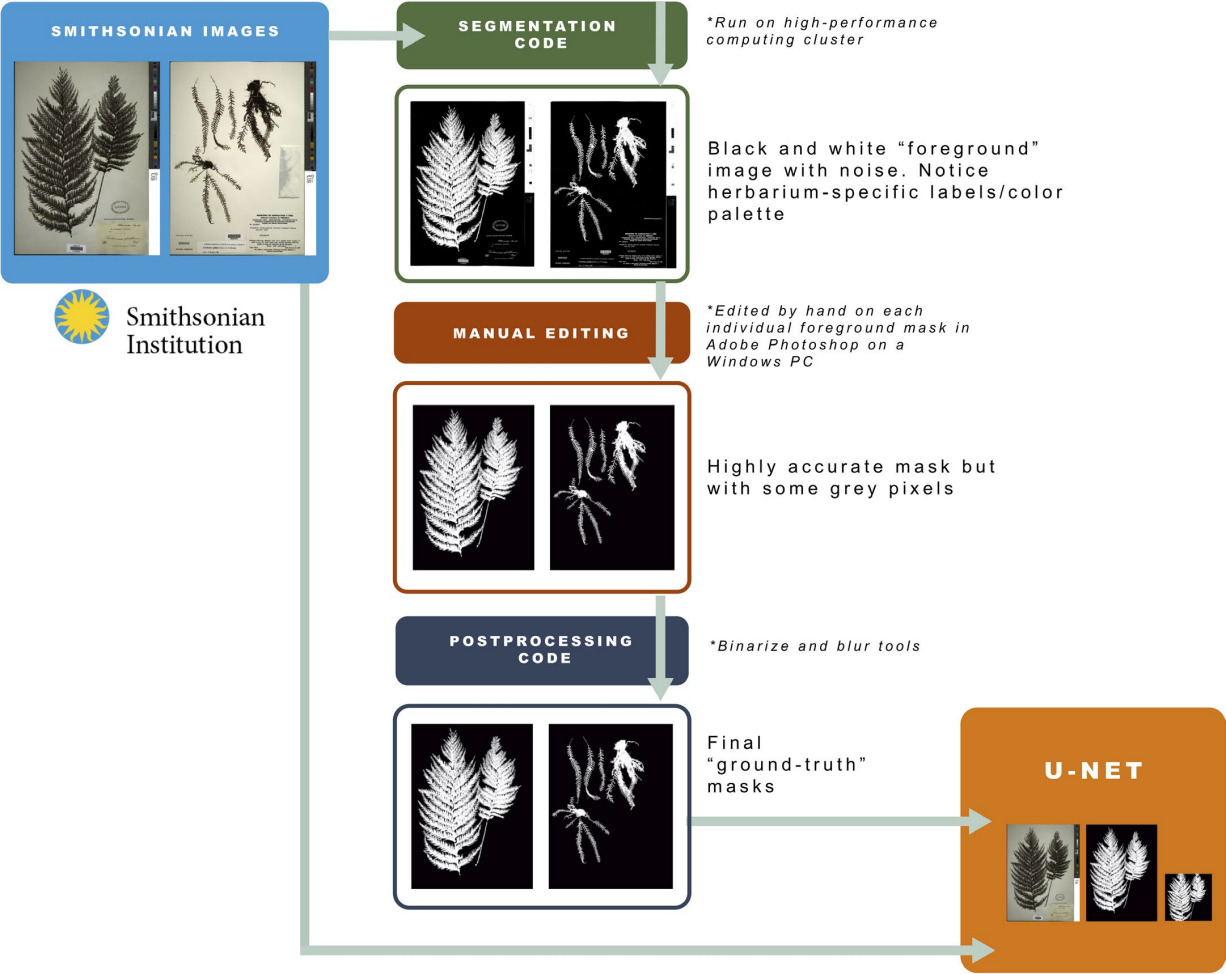
Workflow outlining the automatic and manual steps in generating the image masks and training the U‐Net. High‐resolution JPEG (.jpg) files were exported from the Smithsonian Digital Asset Management System to the High‐Performance Computing Cluster where we ran the segmentation Python code. Outputs from this step were edited in Adobe Photoshop to remove label and color palette before running the postprocessing code (binarize and blur tools) that produced the final ground‐truth masks. These ground‐truth masks were then used as training data for the U‐Net model.

We developed a workflow to generate ground‐truthed herbarium image masks for a simple binary segmentation task in which all pixels are labeled as either plant pixels or background pixels (Fig. 1B). This protocol combines a thresholding method of automatic image segmentation (Otsu, 1979) with manual postprocessing and mask editing to generate high‐resolution and high‐quality image masks (Fig. 2). We describe each component of our workflow below.

First, we import a grayscale version of each original high‐resolution image into Python (van Rossum and Drake, 2009) using the *imread* method in OpenCV (Bradski and Kaehler, 2008). We then use Otsu’s binarization method (Otsu, 1979) as implemented in OpenCV to estimate image thresholds. In short, Otsu’s method searches the distribution of pixel values to estimate a threshold that minimizes intraclass variance. We then assign a binary value (either black [0 in byte image values] or white [255]) to each pixel according to the Otsu threshold to create both foreground and background images. The foreground image is the inverse of the background image. We export both the foreground and background images with filenames corresponding to the original image. We automatically generated these preliminary masks in Python version 3.7 using OpenCV version 4.0.1. The code is available at https://github.com/sidatasciencelab/fern_segmentation.

The ideal image mask would include a single pixel value for plant tissue and another pixel value for the rest of the herbarium sheet, thus allowing the extraction of plant‐only elements for downstream applications. Although the segmentation process described above worked well to include all plant materials in one grouping, each mask also included herbarium elements, such as label data and the color palette (see top middle box, Fig. 2). Because removing these elements automatically is particularly challenging, we instead developed a manual process for their removal. We chose the foreground images (those with white pixels assigned to the plant tissue) and manually edited them in Adobe Photoshop CC 2018 (Adobe Inc., San Jose, California, USA). We used the Brush Tool and the Rectangle Tool to adjust the pixels that should have been assigned a black (0) pixel value during segmentation but were otherwise mislabeled. Due to the behavior of these tools in Photoshop, some of the edge pixels were assigned intermediate gray values, yet pixels needed to be assigned binary pixel values according to our objective. Thus, after we sufficiently edited the extraneous herbarium elements from the images in Photoshop, we binarized the mask images (using the binarize function in OpenCV) to ensure that the pixel values were either 0 or 255.

As a final step in preparing the images for our data set, we utilized the blur method contained within the PlantCV library (Gehan et al., 2017) to remove any remaining disparities (e.g., there were images where dust particles were labeled as plant material and blur uses pixel neighborhood values to smooth away these disparities). For all images, we used three different parameter values and chose the “best” mask by visually inspecting them. This was necessary because the plants vary in size, age, and overall condition (e.g., amount of debris).

### Training a deep learning model for segmentation

**FIGURE 3:**
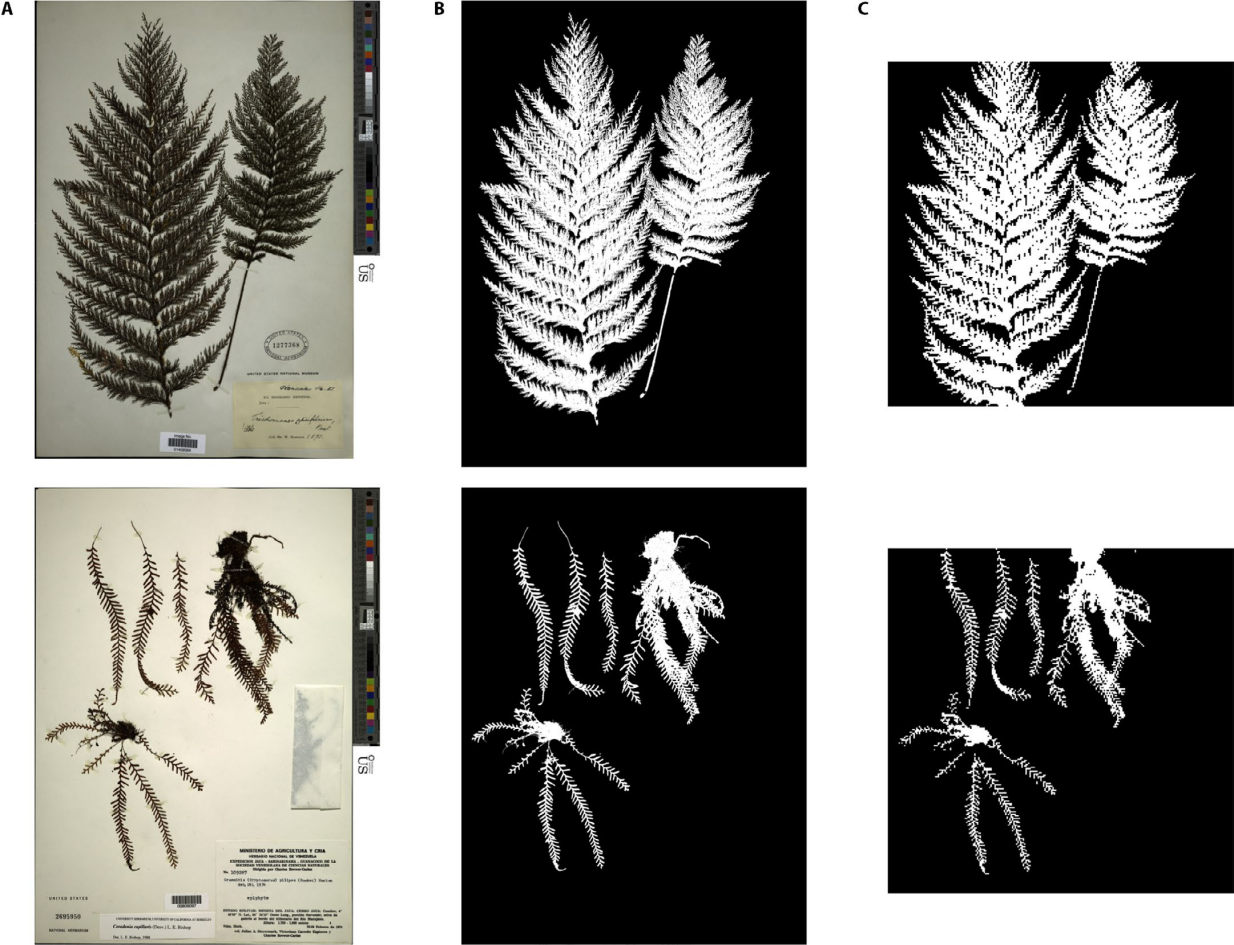
A comparison of high‐resolution original images, ground‐truth masks, and U‐Net‐predicted mask outputs. (A) Two example original images. (B) Ground‐truth masks. (C) Mask outputs predicted by U‐Net (Sørensen–Dice coefficient = 0.95). Note that the predicted masks are all resized to 256 × 256 pixels to maximize downstream model training efficiency regardless of image input size. The square output predictions crop rectangular inputs. Species names from top: *Callistopteris apiifolia* (Presl) Copel.*, Ceradenia capillaris* (Desv.) L. E. Bishop.

We trained a PyTorch (version 1.1.0, Paszke et al., 2017) deep learning model for binary image segmentation of herbarium images using fastai (version 1.0.55, Howard et al., 2018) in Python 3.7. Following an emerging standard of best practices for data set preparation and image transformations (He et al., 2018), we trained a U‐Net style neural network using 80% (*n* = 320) of our original images paired with ground‐truthed masks prepared using the protocol above. This resulted in approximately 21 million pixels with associated class labels for training this model. The goal of such training is to expose the model to a wide diversity of pixel values and contexts paired with the associated pixel class identity (plant or background). All images and associated masks were resized to 256 × 256 pixels to maximize the downstream training efficiency. Our model therefore produces predicted image masks of 256 × 256 pixels regardless of the size of the image input (Fig. 3). The square output predictions crop image inputs if they are rectangular (see Fig. 3C).

We transformed our data randomly during the training process using image augmentation, as is standard practice to maximize the generality of deep learning models. Transformations included flipping images horizontally, rotating (maximum rotation 10 degrees), zooming (maximum zoom by a factor of 1.1), lighting adjustments (maximum adjustment by a factor of 0.2), and warping (maximum warping by a factor of 0.2). All transformations were applied with a probability of 0.75 except for horizontal flipping (probability = 0.5) using fastai.

The model was constructed using the U‐Net architecture (Ronneberger et al., 2015), which is known to be computationally efficient during training and highly accurate for segmentation in a diverse range of applications. Leveraging pretrained models using transfer learning, we used a dynamic U‐Net as implemented in fastai, building our model atop a resnet‐34 architecture pretrained for classification on the ImageNet data set (Deng et al., 2009). The pretrained resnet‐34 architecture was downloaded from the PyTorch model zoo (https://pytorch.org/docs/stable/torchvision/models.html).

We set aside 20% of our original images (*n* = 80) to validate our model. The pixels of these images were never presented to the model during training and thus model performance on these images can be viewed as a measure of the model’s applicability to novel data. We trained our model for 22 epochs (one epoch equals one pass through the training data), following the one‐cycle learning rate policy (Smith, 2018).

### Segmentation model performance

We evaluated the performance of our model by comparing the predicted pixel labels from the U‐Net with the ground‐truth labels we generated for the 80 validation images. Across all 80 images in the validation set, our model achieved a 0.95 Sørensen–Dice coefficient on the predicted masks generated by the model (Fig. 3C). In this case, the Sørensen–Dice coefficient is equivalent to the proportion of shared pixel identities between the ground‐truth mask and the predicted mask. It is important to note that generating predictions for even a single herbarium image involves 65,536 predictions (i.e., 256 × 256 pixels). The performance of our model is therefore evaluated across ~5 million individual pixel predictions.

In order to understand how the model performed across the taxonomic diversity of our sample, we also measured the Sørensen–Dice coefficient for each of the 23 fern families in our validation data (Table 1). We found that the model performed roughly comparably across these groups, although for two families the model generated a Sørensen–Dice coefficient < 0.9. On inspection, the validation images representing these two families (Schizaeaceae and Athyriaceae) expose some key limitations of the model. First, the image representing the Schizaeaceae (catalog number 1054905, see Data Availability) contained very little leaf material and mostly thin stems. In the absence of leaves to outline, the model performance was evaluated entirely on its ability to partition thin stems from the backdrop. In general, our model may struggle with these types of features. The image representing Athyriaceae (catalog number 66902) was generated under less than ideal lighting conditions using older digitization technology and also shows a yellowed herbarium sheet. Model performance may be compromised under these conditions, although the 0.86 Sørensen–Dice coefficient for this image is still likely to meet the standard of quality for nearly all herbarium applications.

**TABLE 1:** Model performance for individual fern families.

Family	No. of validation images	Sørensen–Dice coefficient
Gleicheniaceae	4	0.972
Lygodiaceae	4	0.966
Hymenophyllaceae	13	0.922
Equisetaceae	5	0.917
Ophioglossaceae	4	0.959
Marattiaceae	3	0.971
Psilotaceae	1	0.906
Osmundaceae	1	0.963
Schizaeaceae	2	0.882
Anemiaceae	2	0.952
Cyatheaceae	12	0.968
Polypodiaceae	5	0.948
Dryopteridaceae	4	0.948
Pteridaceae	6	0.963
Tectariaceae	1	0.916
Aspleniaceae	3	0.963
Lindsaeaceae	1	0.936
Blechnaceae	1	0.963
Thelypteridaceae	4	0.954
Athyriaceae	1	0.861
Salviniaceae	1	0.960
Dicksoniaceae	1	0.958
Marsileaceae	1	0.942

## CONCLUSIONS

The deep learning model we present here can rapidly generate high‐quality masks of images of any herbarium sample across the morphological diversity of ferns. Although input images to the model may contain different color bars or palettes and other herbarium‐specific labels and features, the masks retain only the pixels of each image that belong to plant material, meaning the output masks can be combined into a single data set to allow computation across images from multiple herbaria. Our training data set was restricted to ferns; therefore, our model may not work as well for plant tissues absent from our data set (e.g., flowers and fruits). However, the workflow we present is general to herbarium images broadly and can be used by botanists specializing in other plant taxa to create their own set of masks with which to train similar deep learning models for segmentation. We hope that members of the botanical community with interests in specific taxa or tissues will create and share similar high‐resolution data sets and models.

There are ~18.9 million digitized herbarium images currently accessible through the Integrated Digitized Biocollections portal (iDigBio, https://www.idigbio.org/portal). This new scale of herbarium science requires modern tools and novel approaches to wield such massive data. Although automated segmentation is a large part of the preprocessing needed before a data set of herbarium images can be used for downstream deep learning applications, there are other concerns when using digital images of herbarium specimens not addressed in our workflow. For example, in any large data set, it is common to find multiple specimens in which the plant material is contained solely as fragments in envelopes and is therefore not visible. Deep learning may yet offer solutions to these challenges; however, workflows, training data, and models designed for preprocessing herbarium images for massive‐scale (millions of images) analyses are still needed to make deep learning tools accessible to the broader botanical community. It is critical to recognize the “human in the loop” component of machine learning and its applications in the biodiversity sciences. The extent to which botanists and biologists are willing to become familiar with these advanced computational tools will likely predict the utility of machine learning in botanical sciences as well as the novelty of the insights such tools may reveal.

## Data Availability

Jupyter Notebook and Python script are available on GitHub: https://github.com/sidatasciencelab/fern_segmentation. Original images (https://doi.org/10.25573/data.9922148), masks (https://doi.org/10.25573/data.9922232), and metadata (https://doi.org/10.25573/data.11771004) are available on the Smithsonian Institution Figshare (smithsonian.figshare.com).
